# Requirement for NF-κB in maintenance of molecular and behavioral circadian rhythms in mice

**DOI:** 10.1101/gad.319228.118

**Published:** 2018-11-01

**Authors:** Hee-Kyung Hong, Eleonore Maury, Kathryn Moynihan Ramsey, Mark Perelis, Biliana Marcheva, Chiaki Omura, Yumiko Kobayashi, Denis C. Guttridge, Grant D. Barish, Joseph Bass

**Affiliations:** 1 Department of Medicine, Division of Endocrinology, Metabolism, and Molecular Medicine, Northwestern University Feinberg School of Medicine, Chicago, Illinois 60611, USA;; 2 Department of Neurobiology, Northwestern University, Evanston, Illinois 60208, USA;; 3 Unit of Endocrinology, Diabetes, and Nutrition, Université Catholique de Louvain (UCL), Brussels B-1200, Belgium;; 4 Darby Children's Research Institute, Medical University of South Carolina, Charleston, South Carolina 29425, USA;; 5 Robert H. Lurie Comprehensive Cancer Center, Northwestern University, Chicago, Illinois 60611, USA

**Keywords:** NF-κB, circadian, genomics, high-fat diet, inducible transcription, inflammation

## Abstract

The mammalian circadian clock is encoded by an autoregulatory transcription feedback loop that drives rhythmic behavior and gene expression in the brain and peripheral tissues. Transcriptomic analyses indicate cell type-specific effects of circadian cycles on rhythmic physiology, although how clock cycles respond to environmental stimuli remains incompletely understood. Here, we show that activation of the inducible transcription factor NF-κB in response to inflammatory stimuli leads to marked inhibition of clock repressors, including the *Period*, *Cryptochrome*, and *Rev-erb* genes, within the negative limb. Furthermore, activation of NF-κB relocalizes the clock components CLOCK/BMAL1 genome-wide to sites convergent with those bound by NF-κB, marked by acetylated H3K27, and enriched in RNA polymerase II. Abrogation of NF-κB during adulthood alters the expression of clock repressors, disrupts clock-controlled gene cycles, and impairs rhythmic activity behavior, revealing a role for NF-κB in both unstimulated and activated conditions. Together, these data highlight NF-κB-mediated transcriptional repression of the clock feedback limb as a cause of circadian disruption in response to inflammation.

## Abstract (precis)

In this study, Hong et al. investigate how clock cycles respond to environmental stimuli and show that activation of the inducible transcription factor NF-kB in response to inflammatory stimuli leads to marked inhibition of clock repressors, including the *Period*, *Cryptochrome*, and *Rev-erb* genes. Their findings highlight NF-kB-mediated transcriptional repression of the clock feedback limb as a cause of circadian disruption in response to inflammation.

The mammalian circadian clock network is programmed by a transcription–translation feedback loop comprised of basic helix–loop–helix activators (CLOCK/BMAL1) that induce the transcription of their own repressors (PERIOD [PER]/CRYPTOCHROME [CRY]/REV-ERBs) through binding to E-box elements within the promoters of these factors, thereby synchronizing behavioral and physiological rhythms in anticipation of the rising of the sun. This core clock cycle is amplified downstream through direct activation of genes containing a regulatory D-albumin-binding protein (DBP) motif, mediated by PAR-bZIP transcription factors (TFs; DBP, HLF, and TEF) and the E4BP4 repressor (NFIL3), which generate rhythmic physiological outputs in the brain, endocrine tissue, the liver, and immune cells (Wuarin and Schibler 1990; Fonjallaz et al. 1996; Cho et al. 2012; Yu et al. 2013; Fang et al. 2014; Wang et al. 2017; Yeung et al. 2018). Clock output genes exhibit cell type-specific regulation localized to enhancers enriched in binding sites for lineage-determining factors such as hepatocyte nuclear factor 4 (HNF4) in the liver (Fang et al. 2014) and PDX1 in the pancreas (Perelis et al. 2015), mediated by three-dimensional interactions between rhythmic enhancers and target gene promoters (Mermet et al. 2018; Yeung et al. 2018). Furthermore, de novo global run-on sequencing analyses have shown that phase-specific circadian transcription can be attributed to alternating cycles of gene activation and repression in a time of day-dependent sequence (Fang et al. 2014) in parallel with rhythmic recruitment of histone- and DNA-modifying enzymes (Koike et al. 2012; Valekunja et al. 2013; Kim et al. 2014).

Intrinsic clock-coupled transcription cycles play a key role not only within the brain in the regulation of sleep/wake and fasting/feeding rhythms but also throughout most peripheral organs and hematopoietic cells, where they integrate tissue functions important in metabolic, immune, and endocrine processes (Bass and Lazar 2016). Unlike transcriptional oscillations in pacemaker neurons, however, peripheral cell clocks can rapidly become desynchronized from one another in the absence of entraining signals (Hughes et al. 2009). Misalignment between rhythmic feeding cycles driven by the central clock as well as gene expression driven by peripheral clocks results in disorganization in the synchrony between neuroendocrine and peripheral tissue rhythms and has been associated with conditions such as inflammation and obesity (Damiola et al. 2000; Stokkan et al. 2001). However, how environmental signals impact core clock factors and gene oscillations remains incompletely known.

Circadian regulation of immunity and the inflammatory response has been well documented (Gibbs et al. 2012; Spengler et al. 2012; Curtis et al. 2015); conversely, phenotype-driven siRNA screens have identified the inducible transcription factor NF-κB, a master regulator of immune and inflammatory responses (Zhang et al. 2017), as a regulator of the core clock (Zhang et al. 2009). Our present studies were prompted by evidence for NF-κB as a mediator of inducible transcriptional responses to diverse pathogenic stimuli, with evidence for additional endogenous NF-κB functions in neuronal activity, including learning and memory (Meffert et al. 2003), cell survival (Beg et al. 1995; Li et al. 1999b), and circadian signaling (Zhang et al. 2009). While clock genes operate in many metabolic (Andrews et al. 2010; Perelis et al. 2015) and immune (Yu et al. 2013) tissues, genome-wide regulation of clock cycles has been best characterized in the context of liver metabolism (Hughes et al. 2012; Koike et al. 2012; Vollmers et al. 2012). As such, we hypothesized that analyses of the transcriptional effects of NF-κB in the liver would yield insight into mechanisms of environmental disruption across multiple tissues. Here we demonstrate that NF-κB participates in circadian function in unstimulated cells and that its activation repositions CLOCK/BMAL1 genome-wide to sites colocalized with NF-κB, leading to inhibition of core clock repressors and revealing a mechanism by which immune activation can alter circadian rhythms in mice.

## Results

### Stimulation of NF-κB activity inhibits the expression of core clock repressors

**Figure 1.**
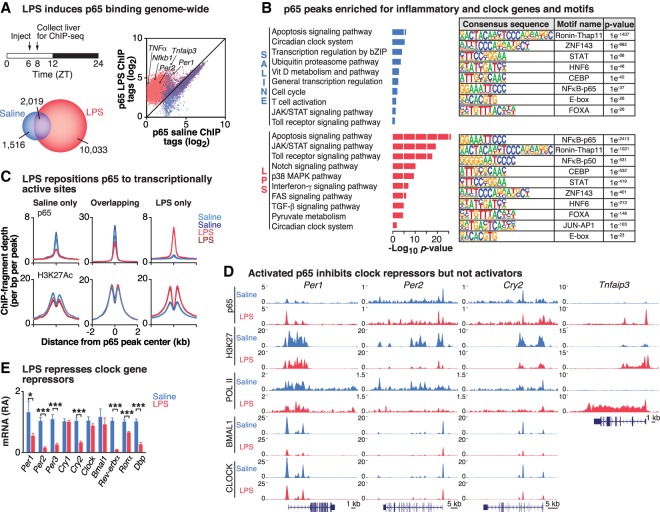
LPS-induced NF-κB transcriptional activation inhibits the repressor arm of the clock. (*A*) Schematic showing LPS injection and tissue collection times. Venn diagram depicting the number of p65 peaks identified in the LPS-stimulated (red) or saline-stimulated (blue) condition (*f* > 1.5, *q* < 0.10) in livers from wild-type mice. Scatter plot depicting log transformed p65 ChIP-seq tag densities for all p65 peaks identified in saline- and LPS-stimulated conditions in wild-type mice. Points *above* the 45° line represent sites inducibly bound by p65, while points *below* the line are peaks with diminished p65 occupancy. *n* = 2 per condition. (*B*, *left* panels) Functional pathway analysis (Panther) of the sites bound by p65 in the saline- and LPS-stimulated conditions identify enriched functional pathways in each condition. (*Right* panels) The top known HOMER motifs enriched at p65-binding sites from ChIP-seq analysis in saline- and LPS-stimulated livers. (*C*) Histograms indicate the occurrence of p65 (*top* row) and H3K27ac (*bottom* row) peaks across 2 kb centered at all p65 peak centers in (1) saline-only (i.e., p65 peaks present only in saline-treated samples, representing p65 peaks “lost” after LPS), (2) overlapping (i.e., p65 peaks present in both saline and LPS conditions), and (3) LPS-only (i.e., p65 peaks unique to LPS-treated livers). (*D*) Representative University of California at Santa Cruz (UCSC) genome browser images of p65, H3K27ac, Pol II, BMAL1, and CLOCK ChIP-seq tracks at clock repressors *Per1*, *Per2*, and *Cry2* and the known p65 target *Tnfaip3*. Normalized tag counts are indicated on the *Y*-axis, and, for each antibody, maximum track height is the same for all conditions. The orientation for each gene is indicated *below* each browser track. *n* = 2 per condition per antibody. (*E*) Quantitative RT–PCR analysis of the panel of circadian clock genes in the liver following either saline or LPS treatment. *n* = 8–10 per group. (*) *P* ≤ 0.05; (***) *P* ≤ 0.001. See also Supplemental Figures S1 and S2.

The circadian clock is composed of transcriptional activators and repressors that generate periodic cycles of behavior and physiology that can be modulated in response to changes in the environment, including inflammation (Marpegan et al. 2005; Okada et al. 2008; Curtis et al. 2015) and high-fat diet (HFD) in mice (Kohsaka et al. 2007; Eckel-Mahan et al. 2013). Since both inflammation and diet are known to stimulate activity of the inducible TF NF-κB, we first sought to examine how NF-κB stimulation following administration of lipopolysaccharide (LPS), a bacterial endotoxin that stimulates the inflammatory response, would acutely impact core clock oscillation and clock-controlled sites genome-wide. To do so, we first treated wild-type mice with either saline or 20 mg/kg LPS (Spengler et al. 2012; Curtis et al. 2015) at Zeitgeber time 6 (ZT6) and performed chromatin immunoprecipitation and sequencing (ChIP-seq) of p65 (a NF-κB subunit), CLOCK, BMAL1, acetylated H3K27 (H3K27ac), and total RNA polymerase II (Pol II) in the liver at ZT8, the zenith of NF-κB activity during acute infection and also a peak time of BMAL1 binding in the liver (Fig. 1A; Rey et al. 2011; Spengler et al. 2012). We next identified peaks that were enriched with each antibody compared with input chromatin samples and assessed differential enrichment over input across all replicates (*n* = 2 for each condition) using DESeq2 (Supplemental Fig. S1A; Zhang et al. 2008). To normalize for differences in sequencing depth, each ChIP library was rescaled by the total number of mapped tags in each library (see the Materials and Methods). LPS strongly induced p65 binding genome-wide (log_2_ fold change over input [*f*] > 1.5; false discovery rate [FDR]-adjusted *P*-value < 0.10), with 12,052 peaks identified in the LPS condition compared with 3535 peaks in the saline condition (Fig. 1A). Functional pathway analysis using protein analysis through evolutionary relationships (Panther) of the sites bound by p65 revealed that LPS treatment resulted in the enrichment of factors within pathways involved in cell survival and inflammation, including the JAK/STAT, Toll receptor, and interferon-γ signaling pathways, while gene ontology terms enriched in saline treatment included basic cellular processes such as transcriptional regulation (Fig. 1B, left panels). Unexpectedly, ontology analysis also indicated that NF-κB-binding sites were enriched for genes involved in the circadian clock system in both the saline- and LPS-stimulated states, suggesting that NF-κB not only affects pathogen response transcriptional networks but also regulates regions of the genome involved in the core clock network in the basal state (Fig. 1B, left panels). As discussed below, the finding that NF-κB colocalizes to sites occupied by CLOCK/BMAL1 in its basal state is consistent with the observation that in the unstimulated condition, NF-κB participates in the establishment of neuronal networks in the hippocampus (Meffert et al. 2003) and the survival of hepatic parenchymal cells (Beg et al. 1995; Li et al. 1999b). Thus, these data are consistent with a role of NF-κB in the regulation of circadian homeostasis both in response to inflammatory challenges and in the unstimulated state. Consistent with the pathway analyses, examination of consensus TF-binding sites in the p65 ChIP-seq peaks in saline and following LPS treatment revealed an expected enrichment of NF-κB motifs in the regions surrounding the peak binding sites as well as an overrepresentation of the canonical E-box motif (CACGTG), a DNA response element bound by CLOCK/BMAL1 (Fig. 1B, right panels; Supplemental Fig. S1B; Ueda et al. 2005; Yoo et al. 2005). These observations further suggest convergence in transcriptional regulation by the circadian clock and NF-κB. Of note, the finding that NF-κB stimulation leads to higher peak density of p65 localized specifically to E-box elements rather than REV-ERB/ROR response elements (RREs) suggests that inflammation may interfere with transcription within the negative limb of the clock, since the E-box element is present in the promoters of clock repressors (Yoo et al. 2005; Cho et al. 2012; Fang et al. 2014).

We further observed three categories of binding events for p65 when comparing saline and LPS conditions: (1) peaks that were present only in the LPS-treated samples (LPS-only), representing “new” p65-binding peaks (10,033 sites); (2) peaks that were present in both the LPS- and saline-treated samples (overlapping), representing sites where the location of p65 binding was “unchanged” following LPS, although the amplitude of peak binding may have changed (2019 sites); and (3) peaks that were present only in the saline-treated samples (saline-only), representing sites where p65 binding was “lost” following LPS stimulation (1516 sites) (Fig. 1A,C; Supplemental Fig. S1C). Of the “new” p65-binding peaks, the most highly enriched sites included known inflammatory targets of NF-κB such as *Tnfaip3* and *Nfkb2* (Fig. 1D; Supplemental Fig. S1D). Overall, these new LPS-induced p65 sites corresponded with increased H3K27ac, a marker of transcriptionally active chromatin (Creyghton et al. 2010), while “unchanged” and “lost” p65 peaks following LPS corresponded with unaltered and reduced H3K27ac, respectively (Fig. 1C; Supplemental Fig. S1C). These data suggest not only that LPS results in a gain of new binding sites for p65 but that there is also a significant redistribution of p65 genome-wide to enable inducible transcriptional regulation in response to environmental stimuli.

Given the emergence of the circadian clock system as a target of NF-κB based on both the pathway and motif analyses, we next examined how p65 regulates the core clock by visualizing p65 binding to promoter regions of specific core clock genes using the University of California at Santa Cruz (UCSC) genome browser in parallel with CLOCK, BMAL1, H3K27ac, and RNA Pol II binding at these same sites in both the saline and LPS conditions (Fig. 1D; Supplemental Fig. S1D). We observed, for example, pronounced p65 binding in saline-treated livers in the untranslated first exon of the *Per2* gene in a region containing both a NF-κB-binding motif (GGGRNYYYCC, where R is a purine, Y is a pyrimidine, and N is any nucleotide) and the noncanonical E2-box (CACGTT) motif (∼220 base pairs [bp] downstream from the NF-κB motif) bound by CLOCK and BMAL1 that has been described previously to preferentially drive circadian transcription of the *Per2* locus (Supplemental Fig. S2A; Yoo et al. 2005). Of note, the fact that we observed colocalization of p65 and CLOCK/BMAL1 within the E2-box region in the *Per2* promoter suggests that their binding is not mutually exclusive. Similarly, we observed p65 binding to the promoters of other core clock genes (*Per1*, *Cry2*, *Dbp*, and *Rev-erb*α) in the unstimulated state (Fig. 1D; Supplemental Fig. S1D), consistent with a tonic role for NF-κB in the control of the negative feedback arm of the clock transcription loop. Interestingly, following LPS treatment, we observed significantly increased p65 binding to the core clock repressor genes (Fig. 1D; Supplemental Fig. S1D). For example, a new p65 peak emerged within the first intron of the *Per2* gene, corresponding with a putative NF-κB-binding motif (log_2_ fold increase over saline = 2.12-fold, *P* = 0.001), although p65 binding in the first exon was slightly reduced (log_2_ fold decrease compared with saline = −1.5-fold, *P* = 0.02) (Fig. 1D; Supplemental Fig. S2A). Similarly, p65 binding increased within promoter regions of the other core clock genes encoding the repressor limb of the clock. Most of these cases involved significantly increased enrichment of p65 at sites already bound to in the unstimulated state (i.e., “overlapping” as in Fig. 1C), such as the 1.95-fold (log_2_ fold) increase in p65 binding to the *Per1* promoter compared with saline (*P* < 0.001). Surprisingly, these effects were specific to the genes encoding the repressor arm of the core clock, as there was minimal binding of p65 to the regulatory regions of the activator genes (*Clock* and *Bmal1*) containing RREs in either the unstimulated or LPS-stimulated state (Supplemental Fig. S1D).

Furthermore, the increase in stimulus-induced p65 binding to the promoters of the clock repressors corresponded with significantly decreased levels of chromatin modification with H3K27ac, in contrast to the increased H3K27ac observed at canonical inflammatory NF-κB targets, where p65 binding increased 3.51-fold (log_2_ fold) compared with saline (*P* < 0.0001) (Fig. 1D; Supplemental Fig. S1D). LPS treatment also decreased the occupancy of RNA Pol II at the promoter regions of core clock repressors (Fig. 1D; Supplemental Figs. S1D, S2B; Fuda et al. 2009), consistent with NF-κB inhibiting transcription of CLOCK/BMAL1 target genes. Using in vitro transcription assays, we further found that NF-κB dose-dependently inhibits transcriptional activation of *Per2* by CLOCK/BMAL1. Increasing doses of either the p65 or p50 subunits of NF-κB reduced CLOCK/BMAL1-mediated transcription of *Per2::Luc* transcription in HepG2 cells transfected with the E2-box of *Per2* fused to Luciferase (Supplemental Fig. S2C; Yoo et al. 2005). Together, these data suggest that LPS-induced p65 binding specifically inhibits transcription of factors within the repressor limb of the core clock (Fig. 1D; Supplemental Figs. S1, S2). It is interesting to note that the inhibitory effect of p65 on the core clock repressors is distinct from the more general mode of activation following LPS exposure (Fig. 1D), since LPS-activated “new” peaks corresponded with increased, rather than reduced, H3K27ac and Pol II activity (Fig. 1C). To confirm these findings, we performed quantitative PCR in livers from LPS- and saline-treated mice and, consistent with the ChIP-seq results, observed significantly reduced expression of the core clock repressors, but not activators, 2 h following LPS injection (Fig. 1E). Interestingly, we also found that LPS resulted in the continued repression of *Per2*, *Per3*, and *Cry1* over the course of 24 h following LPS injection, while *Per1* and *Cry2* increased expression compared with saline-injected mice at the later time points, suggesting that while LPS causes an immediate inhibition of genes containing an E-box regulatory motif within the repressor arm of the clock within the first 2 h, the longer-term impact of clock repressor activity throughout the day appears to be clock gene-dependent (Supplemental Fig. S2D). Overall, the genome-wide analyses support the hypothesis that NF-κB interferes with circadian transcription through inhibition of the clock repressor arm.

### Stimulation of NF-κB relocalizes the occupancy of CLOCK/BMAL1 genome-wide

**Figure 2.**
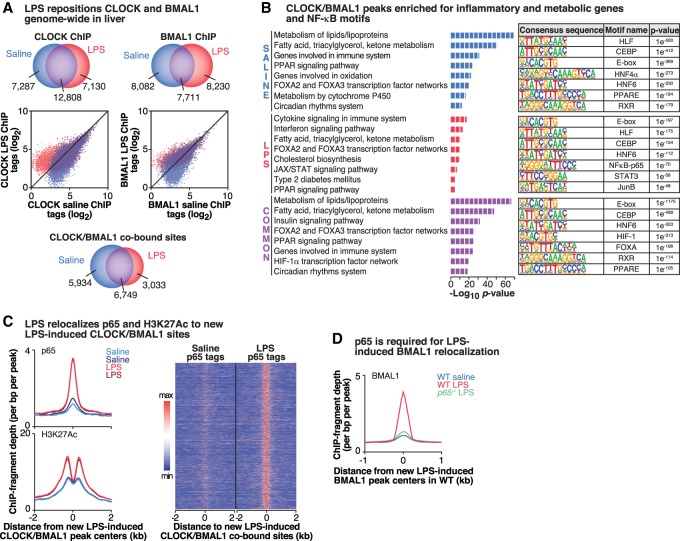
Activation of NF-κB transcription repositions CLOCK/BMAL1 binding genome-wide. (*A*, *top*) Venn diagrams depicting the number of CLOCK- and BMAL1-binding peaks in each condition. (*Middle*) Scatter plots depicting log transformed CLOCK and BMAL1 ChIP-seq tag densities. Points *above* the 45° line represent sites inducibly bound by CLOCK or BMAL1 following LPS stimulation, while points *below* the line are peaks with diminished CLOCK or BMAL1 occupancy following LPS stimulation. (*Bottom*) Venn diagrams using only the CLOCK/BMAL1-cobound sites. *n* = 2 per condition per antibody. (*B*, *left* panels) Functional pathway analysis (Panther) of the sites cobound by CLOCK/BMAL1 in saline- and LPS-stimulated conditions identified enriched functional pathways in each condition. (*Right* panels) The top known HOMER motifs enriched at CLOCK/BMAL1-binding sites from ChIP-seq analysis in saline- and LPS-stimulated livers. (*C*) Histograms representing the occurrence of p65 (*top*) and H3K27ac (*bottom*) peaks within 2 kb of new LPS-induced CLOCK/BMAL1 peak centers. Heat map comparing binding of p65 within 2-kb windows surrounding new LPS-induced CLOCK/BMAL1-cobound peaks following either saline or LPS stimulation. (*D*) Histogram representing the occurrence of BMAL1 peaks within 1 kb of new LPS-induced BMAL1 peak centers in wild type. *n* = 2 per condition. See also Supplemental Figure S2.

While we showed that LPS leads to increased binding of NF-κB to both core clock and inflammatory gene targets (resulting in either gene repression or activation, respectively), we next sought to determine whether inflammatory stimuli impacted the genome-wide binding of the core clock TF activators CLOCK and BMAL1 themselves using previously validated specific antibodies (Perelis et al. 2015). Interestingly, we found that LPS caused a genome-wide relocalization of CLOCK binding: While the total number of CLOCK peaks identified was similar between LPS (19,938) and saline (20,095), only ∼64% (12,808) of these peaks overlapped between the two conditions, there were 7130 unique binding sites in the LPS-stimulated condition (representing new CLOCK-binding sites), and a similar number (7287) of binding sites were present only in the saline condition (representing sites where CLOCK binding was displaced following LPS) (Fig. 2A, left panels). ChIP-seq with BMAL1 following LPS treatment similarly revealed a redistribution of BMAL1 binding (Fig. 2A, right panels). Because we were interested in how environmental stimuli impact the core clock network, we chose to specifically look at sites that were co-occupied by both CLOCK and BMAL1 for all subsequent analyses (Fig. 2A, bottom panel). Surprisingly, LPS stimulation caused CLOCK/BMAL1 to relocalize to sites in proximity to genes involved in the immune system response, interferon signaling, apoptotic, and metabolic signaling pathways (Fig. 2B), suggesting a role of the core clock in the control of inflammatory gene regulation in the liver and consistent with a recent report that BMAL1 regulates inflammatory responses in macrophages by regulating the epigenetic states of enhancers (Oishi et al. 2017). Of particular note, motif analyses revealed significant enrichment in NF-κB-binding motifs at the new LPS-induced CLOCK/BMAL1-binding sites (Fig. 2B), suggesting that CLOCK/BMAL1 recruitment and colocalization with p65 are dependent on LPS induction. Consistent with motif analyses, we observed that acute LPS stimulation induces the relocalization of CLOCK/BMAL1 to sites associated with increased p65 binding, indicating that CLOCK/BMAL1 and p65 redistribute in close proximity to one another (Fig. 2C). To demonstrate that colocalization of p65 with CLOCK/BMAL1 is specific and not a “random” event, we also included a negative control for a motif (LXR, which is highly expressed in the liver and involved in lipid metabolism) that does not enrich with p65 following LPS stimulation (Supplemental Fig. S2E). Furthermore, these sites correspond with increased H3K27ac occupancy in the LPS-stimulated condition (Fig. 2C), revealing that the inducible NF-κB activation at novel CLOCK/BMAL1 sites is associated with increased transcription of new genes in response to the inflammatory stimuli. Thus, LPS repositions CLOCK and BMAL1 to sites convergent with those that undergo inducible binding by NF-κB during activation of inflammatory transcription. Importantly, these findings are consistent with previous reports that p65 acts as a pioneering TF that makes chromatin accessible to cognate transcriptional regulators, potentially leading to the opening of new sites for CLOCK/BMAL1 binding (Ostuni et al. 2013). To determine whether relocalization of CLOCK/BMAL1 to new sites following LPS stimulation required intact NF-κB signaling, we performed BMAL1 ChIP-seq in LPS-treated wild-type versus *p65-*deficient mouse embryonic fibroblasts (MEFs). Intriguingly, we observed that the majority of the new LPS-induced BMAL1 peaks in wild-type MEFs was lost in the LPS-treated *p65* knockout MEFs, suggesting that p65 is required for the genome-wide relocalization of CLOCK/BMAL1 following acute inflammation (Fig. 2D).

### Genetic analysis of NF-κB function in circadian transcription and behavior in vivo

**Figure 3.**
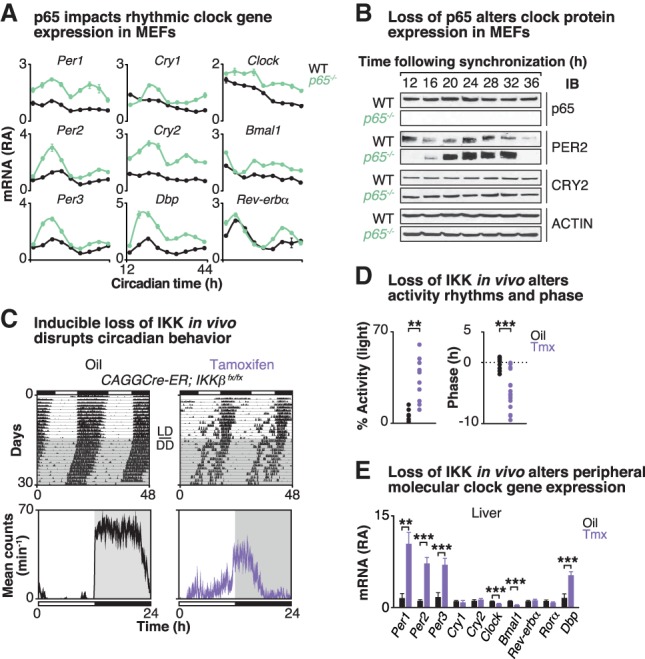
Loss of *p65/IKKβ* disrupts molecular clock expression and behavioral rhythms. (*A*) Quantitative RT–PCR analysis of rhythmic expression of core clock genes in forskolin-synchronized wild-type MEFs (black) compared with *p65* knockout (green) MEFs. Cells were harvested at 4-h intervals for a full circadian cycle, starting 12 h following forskolin synchronization. Data are represented as mean ± SEM. *n* = 2–3 independent experiments, each with four samples per experiment. *P* ≤ 0.001, two-way ANOVA between wild-type and *p65* knockout MEFs for all clock genes shown. (*B*) Representative Western blot analysis (*n* = 2–3) of expression of core clock proteins in forskolin-synchronized wild-type MEFs compared with *p65* knockout MEFs collected at 4-h intervals as above. (*C*, *top* panels) Representative actograms showing wheel-running activity from *CAGGCre-ER;IKKβ*^*fx/fx*^ mice (>2 mo old). Mice were first injected with either oil or tamoxifen once per day for five consecutive days and then maintained on a 12:12 light:dark (LD) cycle in wheel cages for 15 d prior to release to constant darkness (DD). (*Bottom* panels) Representative locomotor activity profiles of *CAGGCre-ER;IKKβ*^*fx/fx*^ mice that received either oil (black) or tamoxifen (purple) are shown. (*D*) The percentage of total activity occurring in the light period (*left*; *n* = 6–10) and the phase angle of entrainment relative to the time of lights off (*right*; *n* = 12–16) are shown. (*E*) Quantitative RT–PCR analysis of core clock gene expression in the liver at ZT4. Values are displayed as relative abundance compared with values of oil-treated mice after normalization to *Gapdh*. *n* = 10–11. Data are represented as mean ± SEM. (**) *P* ≤ 0.01; (***) *P* ≤ 0.001, unpaired *t*-test. See also Supplemental Figure S3.

Given the impact of activated NF-κB on both core clock gene expression and genome-wide CLOCK/BMAL1 occupancy, we next evaluated the impact of loss of NF-κB on clock function in cells by examining the 24-h expression patterns of core clock genes in synchronized wild-type versus *p65-*deficient MEFs (Fig. 3A). Consistent with the p65 ChIP-seq studies, we found that loss of *p65* primarily impacted expression of clock repressors, as we observed increased expression of the *Per* and *Cry* genes in addition to the known clock target gene *Dbp* (Fig. 3A). PER2 and CRY2 protein levels were also significantly increased in synchronized *p65* knockout MEFs (Fig. 3B). Gain-of-function experiments indicated that constitutively elevated p65 is sufficient to inhibit the increased expression of the core clock repressors, since lentivirus-mediated overexpression of wild-type *p65* in *p65* knockout MEFs (i.e., *p65-*rescued MEFs) restored *Per*, *Cry*, and *Dbp* levels to those of wild-type MEFs (Supplemental Fig. S3A).

To next understand how NF-κB regulates circadian behavioral and molecular rhythms in vivo, we generated animals with conditional inactivation of the NF-κB regulatory kinase *IKKβ* in the CNS and peripheral tissues of adult mice using a tamoxifen-inducible allele to overcome perinatal lethality (Fig. 3C,D; Supplemental Fig. S3). We examined wheel-running behavior in oil- and tamoxifen-treated mice expressing a conditional *CAGGCre-ER* allele and the floxed *IKKβ* allele (*CAGGCre-ER;IKKβ*^*fx/fx*^ mice) (Arkan et al. 2005) compared with controls (wild-type, *CAGGCre-ER*, and *IKKβ*^*fx/fx*^ mice) (Fig. 3C,D; Supplemental Fig. S3C–E). While the oil- and tamoxifen-treated control mice displayed wheel-running activity behavior similar to that of oil-treated *IKKβ* mutant mice, tamoxifen-treated *IKKβ* mutant mice displayed significantly increased daytime activity (33.43% ± 4.27% vs. 6.76% ± 1.43%, *P* = 0.002), a shorter free-running period (23.52 h ± 0.05 h vs. 23.68 h ± 0.05 h, *P* < 0.05), a disrupted phase angle of entrainment (activity onset relative to dark onset; −4.77 h ± 0.75 h vs. −0.05 h ± 0.26 h, *P* < 0.0001), and reduced body weight compared with mice carrying the intact *IKKβ* gene (Fig. 3C,D; Supplemental Fig. S3B–E). Importantly, these data further demonstrate that IKK signaling is necessary for maintenance of normal daily activity rhythms even in the unstimulated state. Finally, on a molecular level, we found that in *IKKβ*-deleted mice, repressors of the circadian core genes (*Per1*, *Per2*, and *Per3*) were significantly up-regulated (10-fold to 18-fold increase) in both the liver and white adipose tissue (WAT), but less so in the hypothalamus, compared with tissues from control *IKKβ*-intact mice at ZT4 (Fig. 3E; Supplemental Fig. S3F,G), similar to clock gene expression observed in *p65* knockout MEFs (Fig. 3A; Supplemental Fig. S3). We also observed elevated levels of *Dbp* and reduced levels of *E4bp4* (PAR bZIP TFs) in the livers of the *IKKβ*-deleted mice, consistent with reduced *Dbp* and increased *E4bp4* following LPS, indicating that changes in p65 occupancy reflected regulation at these loci in vivo (Supplemental Fig. S3H,I). Together, these mice provide the first genetic evidence to demonstrate that IKKβ/NF-κB regulates circadian behavior and clock gene expression in vivo to maintain circadian homeostasis during adult life.

### HFD relocalizes NF-κB to chromatin neighborhoods similar to CLOCK/BMAL1

**Figure 4.**
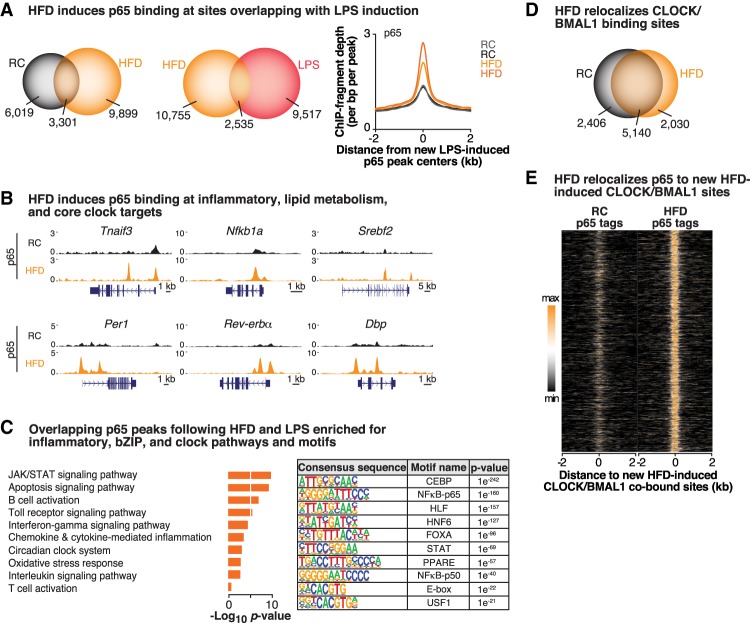
A HFD repositions NF-κB and CLOCK/BMAL to similar chromatin neighborhoods. (*A*) Venn diagram depicting the number of p65 peaks identified in the HFD (orange) versus RC (gray) conditions (*f* > 1.5, *q* < 0.10; *left*) and in the HFD (orange) versus LPS (red) conditions (*middle*) in the liver. (*Right*) Histogram representing the occurrence of HFD-diet induced p65 peaks within 2 kb of LPS-induced p65 peak centers. *n* = 2 per condition. (*B*) Representative UCSC genome browser images of p65 ChIP-seq tracks at known p65 targets, including *Tnfaip3* and *NFκB1a*, as well as at the core clock gene *Per1* and lipid metabolism gene *Srebf2.* Normalized tag counts are indicated on the *Y*-axis, and orientation for each gene is indicated *below* each browser track. (*C*) Functional pathway (Panther) and motif (HOMER) analyses of the sites bound by p65 in both the HFD and LPS conditions identify enriched functional pathways and binding motifs in each condition. (*D*) Venn diagram depicting the number of CLOCK/BMAL1 peaks identified in the RC-fed versus HFD-fed condition (*f* > 1.5, *q* < 0.10) in livers from wild-type mice. *n* = 2 per condition per antibody. (*E*) Heat map comparing binding of p65 in RC-fed and HFD-fed conditions within 2-kb windows surrounding new HFD-induced CLOCK/BMAL1-cobound peaks following a HFD. See also Supplemental Figure S4.

Mounting evidence from work in mice has demonstrated that many of the complications of overnutrition, including cardiovascular disease, appetitive dysregulation, and insulin resistance, result in part from a state of chronic low-grade inflammation induced by activation of IKK signaling and NF-κB-mediated transcription within both the hypothalamus and peripheral tissues such as the liver and fat (Arkan et al. 2005). Since we observed that inducible NF-κB signaling following acute inflammatory stimuli (i.e., LPS) impacts the transcriptional dynamics of the circadian clock (Figs. 1, 2) and that a HFD disrupts the circadian clock system (Kohsaka et al. 2007; Eckel-Mahan et al. 2013), we sought to determine whether a HFD might similarly impact the circadian clock system through relocalization of both NF-κB and CLOCK/BMAL1 binding genome-wide in peripheral tissues. We therefore fed wild-type C57BL6/J mice either a 45% custom-made HFD (Research Diets, D06022405) or regular chow (RC) (Harlan Teklad, 7012) for 4 wk prior to performing ChIP-seq for p65 in the liver at ZT8, the zenith of CLOCK/BMAL1 binding (Fang et al. 2014). We first identified p65 peaks that were consistent and statistically enriched across all replicates (*f* > 1.5, FDR-adjusted *P*-value < 0.10) and observed that a HFD led to an increase in p65 binding genome-wide, with 13,200 peaks following a HFD compared with 9320 peaks in the RC condition, demonstrating inducible recruitment of NF-κB in response to macronutrient stimuli (Fig. 4A). Remarkably, a HFD leads to increased p65 occupancy at sites similarly induced by LPS, as nearly 21% (*P* < 0.0001, two-way ANOVA) of the sites that p65 inducibly binds to following acute LPS stimulation overlapped with sites bound by p65 following chronic HFD treatment (Fig. 4A), including genes involved in immune system and NF-κB activation such as *Tnfaip3* and *Nfkb1a* (Figs. 1D, 4B; Supplemental Fig. S1A), indicating that a HFD induces a significantly increased inflammatory signature compared with RC. Furthermore, p65 binding increased at core clock repressor genes, including *Per1*, *Rev-erb*α, and *Dbp* (Fig. 4B), but not at promoters of genes encoding the clock activators *Clock* and *Bmal1* (Supplemental Fig. S4A). Of note, Panther pathway analyses of these overlapping sites show enrichment of inflammatory pathways, including JAK/STAT, Toll receptor, and chemokine/cytokine signaling pathways, all consistent with inducible NF-κB activation, and, importantly, the circadian clock system emerges as a common pathway regulated by p65 binding following both acute and chronic inflammatory stimuli (Figs. 1B, 4C). Moreover, motif analyses revealed significant enrichment in metabolic and circadian bZIP factors (CEBP and HLF), HNF6-binding motifs, and the circadian clock pathway (E-box and USF1), in addition to p65 (Fig. 4C), following a HFD. To extend these findings, we performed quantitative PCR in both WAT and livers from HFD- and RC-fed mice and observed significantly reduced expression of the core clock repressors in both adipose tissue and livers of HFD-fed mice, consistent with previous reports that a HFD leads to reduced clock gene expression in a tissue-specific manner in peripheral tissues (Supplemental Fig. S4B; Kohsaka et al. 2007; Eckel-Mahan et al. 2013). Finally, a HFD induces a significant number of new p65 sites notably associated with metabolism of lipids, including the SREBPs, which have metabolic regulatory connection to diverse processes such as innate immunity (Im et al. 2011), indicating that NF-κB activation also couples pathways involved in cell type-specific and nutrient-responsive anabolic pathways such as lipogenesis and lipid metabolism as well as cholesterol biosynthesis (Fig. 4B,C; Shao and Espenshade 2012). Together, these data indicate significant overlap between NF-κB activation following both acute and chronic inflammatory challenges in the liver, leading us to next examine the interplay between NF-κB activation and CLOCK/BMAL1 genome-wide binding following a HFD.

To elucidate the impact of a HFD on CLOCK/BMAL1 binding genome-wide, we performed CLOCK and BMAL1 ChIP-seq following HFD treatment. Similar to the findings from LPS treatment, the total number of sites bound by CLOCK/BMAL1 was similar between RC and a HFD (7546 and 7170 peaks, respectively); however, the location of CLOCK/BMAL1-binding sites shifted, as there were 2030 new HFD-specific CLOCK/BMAL1 peaks and 2406 peaks that were present in the RC condition only (representing sites that “lost” CLOCK/BMAL1 binding following a HFD) (Fig. 4D). Consistent with our previous observation that redistribution of CLOCK/BMAL1 throughout the genome is a feature of acute inflammation (LPS)-induced gene transcription (Fig. 2), a HFD similarly redistributes CLOCK/BMAL1 to sites associated with the immune system and lipid and fatty acid metabolism (Supplemental Fig. S4C). Finally, we demonstrate that p65 and CLOCK/BMAL1 are in close proximity to one another following high-fat feeding, as p65 colocalizes with new HFD-induced CLOCK/BMAL1-binding sites following a HFD (Fig. 4E). Together, these data demonstrate that both acute inflammation and chronic inflammation cause the relocalization of the forward limb of the clock to sites convergent with activated NF-κB, H3K27ac, and RNA Pol II in the liver.

## Discussion

### Circadian repressors in the negative feedback arm exhibit a dynamic response to inducible TFs

Circadian components within the core circadian feedback loop were identified based on their autoregulatory activity comprised of forward limb activators that induce their own repressors within the negative limb. A canonical property of the circadian clock is its nearly 24-h periodicity, which enables anticipation of daily changes in the environmental light cycle at the level of both the brain and peripheral tissues. Endogenous clock cycles are in turn synchronized across tissues by oscillating hormonal and molecular signals, the best characterized being those that regulate the expression of repressors within the negative feedback arm. For example, temperature fluctuation in response to sleep/wake and fasting/feeding states provides input into the clock cycle through the activation of heat shock factor 1 and temperature-sensitive RNA-binding proteins and their induction of transcription of the negative arm of the clock (Buhr et al. 2010; Morf et al. 2012). Furthermore, an unbiased TF screen identified the serum response kinase pathway as a novel clock-regulating signal activated in the blood in response to meals and regulating transcription of the negative limb repressors (Gerber et al. 2013). The use of genetic reporters for core clock oscillation further revealed that oxygen, hormones, gut microbes, cytokines, and inflammation signaling pathways also influence both expression of core clock factors and the amplitude and periodicity of transcriptional oscillations (Zhang et al. 2009; Chen et al. 2012; Peek et al. 2017). These observations suggest that the cell-autonomous activity of the clock is coupled to the expression and activity of local inducible TFs.

However, there has been a gap in understanding the mechanisms by which the core clock integrates specific environmental signals at the level of gene transcription and chromatin occupancy and how signal-dependent TFs might relay environmental information to modulate the timing of tissue-level gene oscillations. In this study, we provide evidence that the immune regulatory TF NF-κB is essential for circadian homeostasis and that clock function is particularly vulnerable to direct and indirect triggers of NF-κB activation, establishing a role for NF-κB activity in the circadian response to inflammatory stimuli. Namely, we found that the inflammation signaling TF NF-κB antagonizes transcription of CLOCK/BMAL1 target genes by directly binding to the promoters of the core clock repressors PER and CRY (i.e., the negative limb of the clock), resulting in a highly specific transcriptional repression signature at core clock genes; repressing clock output PAR bZIP factor DBP; and activating E4BP4, which may influence circadian organization (Ueda et al. 2005; Ohno et al. 2007). We further found that the activating clock TFs (CLOCK/BMAL) are relocalized genome-wide to sites convergent with those bound by NF-κB and H3K27ac and enriched in Pol II in response to either acute inflammatory insult or a HFD. Together, these data demonstrate that stimuli leading to NF-κB activation result in both direct transcriptional repression of the core clock repressors (but not activators) and repositioning of the clock activator proteins genome-wide, ultimately altering circadian transcriptional dynamics. Last, we established that a basal level of NF-κB expression is crucial for circadian homeostasis, since abrogation of NF-κB signaling in adult mice disrupts circadian behavioral and molecular rhythms, and also observed NF-κB binding at the promoters of the core clock repressors even in the unstimulated state, indicating a role for NF-κB in circadian function even in its latent state of immune signaling.

### Genome-wide analyses reveal collaborative activating or repressive actions of inducible TFs

Genome-wide analyses have identified epigenetic factors important in determining circadian cycles (Duong and Weitz 2014; Tamayo et al. 2015) and how collaborative interactions with inducible TFs contribute to waves of rhythmic transcription within promoter-distal regulatory enhancer elements (Fang et al. 2014; Trott and Menet 2018; Yeung et al. 2018). The rhythmic transcription cycle can be traced to the recruitment of both chromatin- and DNA-modifying enzymes that either activate (e.g., MLL3 and P300/CBP) (Koike et al. 2012; Valekunja et al. 2013) or repress (e.g., HDAC3 and NurRD) (Kim et al. 2014) transcription, resulting in either permissive or nonpermissive local environments. One example of a clock factor in establishing a permissive context for metabolic gene transcription stems from the observation that loss of the clock repressor REV-ERB results in the unopposed derepression of the lineage-determining HNF6 (Zhang et al. 2016). In addition to regulation of hepatic lipogenesis and liver function, REV-ERB rhythmic activity also plays an important role in not only controlling the activation of innate immune cells—as REV-ERBs regulate the response of mouse macrophages to injury through modulation of enhancer activity (Lam et al. 2013; Eichenfield et al. 2016; Oishi et al. 2017)—but also circadian behavior (Cho et al. 2012). Indeed, we observed here that inflammatory stimuli lead to reduced H3K27ac and Pol II binding to the promoter of the clock repressor gene *Rev-Erb*α (Supplemental Fig. S1D), while the HNF6 motif emerged as enriched among p65-binding sites during inflammation (Fig. 1). These data indicate intricate coupling of the extrinsic signal-induced TF NF-κB, the core clock, its metabolic output, and behavioral rhythms that integrate clock cycles with the light–dark environment.

### NF-κB integrates metabolic inflammation and circadian homeostasis at the level of the transcriptome

NF-κB has emerged in recent years as having roles not only in the inflammatory response but also as a mediator of proliferation, apoptosis, neurogenesis, learning and memory behavior, and aging within both peripheral tissues and the CNS (Li et al. 1999a,; Meffert et al. 2003; Zhang et al. 2013). Furthermore, many of the complications of obesity have been shown to correspond with infiltration of inflammatory cells into metabolic tissues, including hypothalamus, adipose, and liver, and stimulation of proinflammatory cytokine signals downstream from NF-κB. The broad range of NF-κB activity in tissues that do not participate in immunity per se and the contribution of NF-κB activation to metabolic disease pathogenesis (Hotamisligil et al. 1993; Cai et al. 2004, 2005; Arkan et al. 2005) raise important questions concerning the genomic effectors of NF-κB activity within the brain, immune tissue, and other peripheral metabolic organs such as the liver. Of note, our comparative analyses of Gene Expression Omnibus (GEO) repository p65 ChIP-seq data for TNFα-stimulated human lymphoblasts, human umbilical vein cells (HUVECs), HeLa B2 cells, and A549 lung carcinoma cells reveal that NF-κB binds to E-box-containing promoter regions of core clock repressors in these cell types as well (Supplemental Fig. S5; Nicodeme et al. 2010; The ENCODE Project Consortium 2011; Rao et al. 2011; Raskatov et al. 2012; Brown et al. 2014), suggesting that NF-κB regulates the clock in metabolic, immune, and other cell types and tissues and that this regulation is conserved from mice to humans. Furthermore, extensive research has shown that immune cell activation with cytokine or Toll-like receptor (TLR) ligand results in reprogramming of canonical enhancer marks, establishing a “permissive” context for the chromatin binding and activity of NF-κB (Kaikkonen et al. 2013; Ostuni et al. 2013). Interestingly, like other inflammatory regulators, NF-κB does not function strictly as either a repressor or activator of gene expression but rather exerts distinct effects on selective targets that are likely determined by chromatin context and gene-dependent features. Our findings here cast new light on NF-κB as a pivotal genomic control node integrating metabolic inflammation and circadian systems at both the cellular and organismal levels. In particular, NF-κB directly regulates the expression of the negative limb of the clock, while, at the same time, the clock activator TFs CLOCK/BMAL1 colocalize with NF-κB at new sites to regulate transcription following inflammatory stimuli.

How adult animals adapt to changes in nutrients and inflammation under normal and pathologic conditions may be influenced by the activation of NF-κB and its modulation of circadian function. However, whether improper NF-κB activation may contribute to metabolic disease and aging in part through dysregulation of circadian systems remains an important question to be explored, particularly in regard to the determination of which neuronal cell types are influenced by NF-κB signaling in the brain. Finally, in the context of recent work interconnecting the clock with immunity, it will be intriguing to test whether NF-κB might participate in clock reprogramming within the intestinal epithelium and other host cells following changes in gut microbiota composition (Mukherji et al. 2013). Indeed, recent work has demonstrated essential roles for the circadian repressors REV-ERBα and E4BP4 in modulating the development and activity of adaptive Th17 cells (Yu et al. 2013) and innate lymphoid cells (Yu et al. 2014), dendritic cells (Wang et al. 2017), and macrophages (Nguyen et al. 2013), which mediate physiological responses to changes in the microbiota and extrinsic immune challenges. Together, these data provide insight into how inducible transcription can regulate circadian homeostasis at both the transcriptional and behavioral levels.

## Materials and methods

### Mice

Male C57BL/6J (8–9 wk old) and *CAGGCre-ER* transgenic mice were purchased from the Jackson Laboratory (stock nos. 000664 and 004682, respectively), and *IKKβ*^*fx/fx*^ mice were obtained from Dr. M. Karin (Arkan et al. 2005). All mice were group-housed under a 12:12-h light:dark (LD) cycle with RC (Harlan Teklad, 7012) and water ad libitum unless noted otherwise. For the LPS experiments, 20 mg of *Escherichia coli* LPS per kilogram of body weight (Sigma-Aldrich, 055:B5) or saline was injected intraperitoneally at ZT6 (i.e., 6 h after lights on, corresponding to the peak of NF-κB activity in the liver), and mice were sacrificed at ZT8 to obtain tissues. For high-fat studies, half of the mice were fed a custom-made HFD (45% kcal from fat; Research Diet, Inc., custom diet no. D06022405) for 4 wk, while the other half remained on RC (HFD-fed mice gained 6.13 g ± 0.56 g compared with RC-fed mice, which gained 1.95 g ± 0.23 g, *P* < 0.001) (Supplemental Table S1). For tamoxifen-inducible mice generated by crossing *CAGGCre-ER* and *IKKβ*^*fx/fx*^ mice, 75 mg of tamoxifen per kilogram of body weight (Sigma) or corn oil was injected intraperitoneally once every 24 h for a total of five consecutive days into 2- to 10-mo-old mice, and behavioral analyses were performed 7 d after injection. Animal care and experimental procedures were in accordance with guidelines of the Northwestern University Institutional Animal Care and Use Committee (IACUC).

### Cell lines

MEFs lacking NF-κB *p65* (gift from Dr. D. Guttridge) (Wang et al. 2009) were grown in Dulbecco's modified Eagle's medium (DMEM) supplemented with 10% fetal bovine serum (FBS). To generate *p65*-rescued MEF cell lines, retrovirus expressing wild-type *p65* cDNA (pBabep65) versus nonexpressing plasmid (provided by Dr. D. Guttridge) was used to infect *p65* knockout MEFs, and stable cell lines were selected with 2 µg/mL puromycin. *p65* knockout and rescued *p65* knockout MEFs were authenticated by quantitative PCR expression of p65. For analysis of rhythms in MEFs, cells were synchronized with 10 µM forskolin (at a time point designated as CT0) for 1 h, and, starting 12 h after the forskolin shock (CT12), cells were collected every 4 h for 24 h for RNA and protein analysis. For ChIP in MEFs, cells were treated with 100 ng/mL LPS or saline for 1 h prior to fixation and nucleus isolation.

### Behavioral analyses

For the tamoxifen-inducible experiments, mice were placed in individual wheel-running cages, and activity was recorded and analyzed using the ClockLab data collection system (Actimetrics). For analyses under entrained (LD) conditions, locomotor activity was measured using ClockLab data analysis software. For analyses in constant darkness (DD), free-running periods were measured by line fitting of activity onsets from data collected during the DD period of the assay using ClockLab data analysis software. The phase angle of entrainment was calculated by extrapolating the estimated activity onset to the last day of the LD cycle and the time of lights off as described previously (Siepka and Takahashi 2005).

### RNA isolation and quantitative real-time PCR

Total RNA was extracted from frozen tissue with Triazol reagent (Molecular Research Center, Inc.). RNA was then reverse-transcribed using the high-capacity cDNA reverse transcription kit (Applied Biosystems). Quantitative real-time PCR was performed with SYBR Green (Applied Biosystems) in an Applied Biosystems 7900HT Fast real-time PCR system (Applied Biosystems). Primer sequences are listed in Supplemental Table S2 (Kohsaka et al. 2007; Tong et al. 2010; Shimomura et al. 2013). Relative expression levels (normalized to *Gapdh*) were determined using the comparative CT method. All ANOVA analysis was performed using GraphPad Prism version 7.0 for Mac OS X (GraphPad Software).

### Western blotting

Tissues and cultured cells were homogenized in a buffer containing 20 mM HEPES (pH 7.5), 100 mM NaCl, 0.05% Triton X-100, 1 mM dithiothreitol (DTT), 1 mM EDTA, and protease inhibitor and phosphatase inhibitor cocktails (Complete mini and phoSTOP phosphatase inhibitor cocktail tablets; Roche Applied Science). Homogenates were cleared by centrifugation at 10,000*g* for 10 min at 4°C, supernatants were collected, and protein concentration was estimated using Bio-Rad DC protein assay (Bio-Rad) according to the manufacturer's instructions. Total protein was resolved on an 8%–10% SDS–polyacrylamide gel by electrophoresis. Thereafter, proteins were electrotransferred onto a nictrocellulose transfer membrane. The membranes were blocked with PBST (PBS with 0.1% Tween-20) containing 5% powdered milk for 1 h. Anti-PER2 and CRY2 antibodies were a generous gift from Dr. C. Lee, anti-β-ACTIN was from Santa Cruz Biotechnology (sc-1616), anti-NFκB p65 was from Abcam (ab7970), and anti-guinea pig IgG secondary antisera horseradish peroxidase was from Jackson ImmunoResearch. Proteins were visualized with a chemiluminescence detection system with subsequent exposure to autoradiographic ﬁlm.

### Luciferase assays

HepG2 cells were transiently transfected with 50 ng of *Clock*, 50 ng of *Bmal1*, and 30, 60, or 90 ng of NF-κB p50- and/or p65-expressing constructs as well as 100 ng of *Per2* firefly luciferase reporter and 1.6 ng of renilla luciferase reporter (control for transfection efficiency) using Lipofectamine 2000 (Invitrogen, 11668-019). Forty-eight hours following transfection, cells were lysed, and luciferase activity was monitored using the dual-luciferase reporter assay system (Promega, E1910). Firefly luciferase values were normalized to renilla luciferase and then normalized to those obtained without *Clock* and *Bmal1* (as described in Ramsey et al. 2009).

### ChIP-seq and data analysis

Liver tissue and MEFS were fixed for 30 min in 2 mM disuccinimidyl glutarate and for 10 min in 1% formaldehyde and then either frozen at −80°C or processed immediately. ChIP assays were performed as described elsewhere (Barish and Tangirala 2013) with modification. Briefly, nuclei were isolated in buffer containing 1% SDS, 10 mM EDTA, 50 mM Tris-HCl (pH 8.0), and protease inhibitors and then sonicated using a Diagenode Bioruptor to shear chromatin into 200- to 1000-bp fragments. Protein–DNA complexes were incubated with antibodies against p65 (Abcam, ab7970; Santa Cruz Biotechnology, sc-372), BMAL1 and CLOCK (Perelis et al. 2015), H3K27ac (Active Motif, 39133), and RNA Pol II (Santa Cruz Biotechnology, sc-899) and immunoprecipitated with IgG paramagnetic beads (Thermo Fisher). Eluted chromatin was isolated using MinElute PCR purification columns (Qiagen). Sequencing libraries were generated using NEBNext Ultra II DNA library preparation kits (New England Biolabs) according to the manufacturer's instructions. The quality of the library and concentration were determined by Bioanlayzer using the high-sensitivity chip (Agilent) and quantitative PCR-based quantification (NEBNext library quantification kit), respectively. Libraries were sequenced using 75-bp single-end reads on an Illumina NextSeq 500 instrument to a depth of >10 million mapped reads.

Results were visualized by preparing custom tracks for the UCSC browser. Raw sequence reads were aligned to the mm10 reference genome using Bowtie version 1.1.1 (Langmead et al. 2009) with parameters “-best” and “-m 1” to ensure reporting of uniquely mapped reads (tags). For visualization of peaks, the MACS version 2.0.10 peak caller (Zhang et al. 2008) was used to obtain bedGraph files, which were subsequently converted to BigWig files for display. Peaks were identified using a FDR of <0.05%, and input chromatin-derived DNAs were used as controls. Aligned sequence reads were normalized to signal per million reads by specifying the “--SPMR” parameter. For H3K27ac and Pol II, “--nomodel” and “--broad” parameters were specified, and for p65, CLOCK, and BMAL1, the default option was used.

Subsequent data analysis was performed using HOMER, a software suite for ChIP-seq analysis (http://homer.ucsd.edu/homer/index.html). Two biological replicates were analyzed for all conditions (i.e., *n* = 2 per condition), and highly significant (*P* < 0.0001) Pearson correlation coefficient values were obtained for each replicate set (*r* = 0.9 and 0.855 for p65 ChIP replicates; *r* = 0.849 and 0.85 for BMAL1 ChIP replicates, and *r* = 0.890 and 0.888 for CLOCK ChIP replicates in saline and LPS conditions, respectively) (Supplemental Fig. S1A). To identify peaks that were both statistically enriched across replicates and differentially enriched across conditions, “getDifferentialPeaksReplicates.pl” and “getDiffExpression.pl,” which uses the R/Bioconductor package DESeq2 (Love et al. 2014) within HOMER, were performed, respectively. The threshold for the number of tags that determine a valid peak was selected for a FDR-adjusted *P*-value of <0.1 with a log_2_ fold change of >1.5-fold enrichment over the input or control sample. Identified peaks were annotated to the nearest transcription start site. Functional annotation of *cis*-regulatory regions was performed using GREAT (McLean et al. 2010).

### Quantification and statistical analyses

Data are represented as mean ± SEM, and statistical significance was determined by unpaired two-tailed Student's *t*-test unless indicated otherwise; a *P*-value of <0.05 was considered significant.

### Accession numbers

ChIP-seq data sets have been deposited in NCBI's GEO with accession number GSE117488.

## Supplementary Material

Supplemental Material
