# Supplementary material for: Requirement for NF-κB in maintenance of molecular and behavioral circadian rhythms in mice
Source: Genes Dev. 2018 Nov 1;32(21-22):1367–79. doi: 10.1101/gad.319228.118 (PMC6217733; doi:10.1101/gad.319228.118)
Supplement: Supplemental Material [file supp_32_21-22_1367__index.html]

Requirement for NF-κB in maintenance of molecular and behavioral circadian rhythms in mice — Supplemental Material 

# Requirement for NF-κB in maintenance of molecular and behavioral circadian rhythms in mice

## Supplemental Material

- Supplemental\_Material.pdf
