# Supplementary material for: Requirement for NF-κB in maintenance of molecular and behavioral circadian rhythms in mice
Source: Genes Dev. 2018 Nov 1;32(21-22):1367–79. doi: 10.1101/gad.319228.118 (PMC6217733; doi:10.1101/gad.319228.118)
Supplement: Supplemental Material [file supp_gad.319228.118_Supplemental_Material.pdf]

SUPPLEMENTAL FIGURE LEGENDS

Figure S1. LPS-induced NF-κB activation represses circadian clock repressors. *(A)* Scatter plots depicting log-transformed ChIP-sequencing tag densities for all p65, BMAL1, and CLOCK peaks identified in each replicate (n=2) in saline- (top) and in LPS- (bottom) treated mice. In each condition, there is significant overlap at the 45-degree line which represents equivalent binding between the replicates. P<0.0001 for all comparisons. *(B)* Table indicating percentage of p65 peaks in both saline and LPS conditions that contained each of the listed consensus motif sequences, as well as the total number of sites in each condition. *(C)* Strength of binding (reads/bp/peak) at p65 sites that were either lost (left), shared (middle), or gained (right) following LPS treatment for p65 (top) and H3K27Ac (bottom) ChIP-seq. *(D)* Overlaid representative UCSC genome browser images of p65, H3K27Ac, POL II, BMAL1, and CLOCK ChIP-seq tracks at core clock genes *Cry1*, *Dbp*, *Rev-erbα*, *Clock*, and *Bmal1*, as well as at the known NF-κB target *Nfkb2*. Normalized tag counts are indicated on the y-axis, and for each antibody, maximum track height is the same for all conditions. Orientation for each gene is indicated below each browser track. (n=2 per condition per Ab).

Figure S2. NF-κB activation acutely represses circadian clock repressors and is enriched at LPS-induced CLOCK/BMAL1 sites. *(A)* Browser tracks of p65, BMAL1, and CLOCK ChIP-seq at the *Per2* promoter, where validated and putative NF-κB (red and black tick marks, respectively) and E-box (green tick mark) binding motifs are indicated below the browser tracks. Sequence analysis using the promoter prediction and regulatory sequence algorithm (http://www.genomatix.de/matinspector.html) identified putative NF-κB binding sites. *(B)*

Representative browser tracks of total POL II ChIP-seq following saline or LPS treatment at the positive control *Pai1* (*plasminogen activator inhibitor-1*) and the negative control *Drd2* (*Dopamine receptor D_2*) gene promoters. *(C)* Relative luciferase activity of PER2-LUCIFERASE in the presence of increasing doses of the NF-κB subunits p65 and p50 in HepG2 cells (n=5-11). *(D)* Time course of clock gene expression in liver following LPS injection. WT mice were injected with LPS at ZT6, followed by tissue collection for qPCR analysis of clock repressors at indicated times post-injection (p<0.0001, two-way ANOVA, for all clock genes shown) (n=3-4). *(E)* Histograms showing enrichment of NF-κB p65 and p50 motifs in comparison with LXRE motifs at LPS-induced compared to saline-treated CLOCK/BMAL1 peak centers. Data are represented as mean ± SEM. *p≤0.05, **p≤0.01, ***p≤0.001 unpaired t test.

Figure S3. Loss of p65/IKK disrupts molecular clock gene expression and behavioral rhythms. *(A)* Quantitative RT-PCR analysis of rhythmic expression of core clock genes in forskolin-synchronized WT MEFs (black) compared to p65 KO (green) and *p65*-rescued (gray) MEFs. Cells were harvested at 4 hr intervals for a full circadian cycle, starting 12 hrs following forskolin synchronization. Data are represented as mean ± SEM (n=2-3 independent experiments, each with 4 samples per experiment) (p<0.0001, two-way ANOVA between WT vs. *p65* KO MEFs and *p65* KO vs. *p65*-rescued MEFS for clock genes shown). *(B)* Change in body weight in *$IKK\beta^{fx/fx}$* and *CAGGCre-ER;$IKK\beta^{fx/fx}$* mice 5 weeks post-injection of either oil or tamoxifen (n=4-15). *(C)* Period distribution shown for oil- or tamoxifen-treated *CAGGCre-ER;$IKK\beta^{fx/fx}$* mice (n=6-9). *(D)* Representative actograms showing wheel-running activity from *CAGGCre-ER; $IKK\beta^{fx/fx}$* and control littermate (*$IKK\beta^{fx/fx}$*, *CAGGCre-ER*, and WT) mice which received either oil (black) or tamoxifen (purple). Mice were injected i.p. at 75 mg/kg body weight of tamoxifen (prepared in

corn oil) once every 24 hrs for a total of 5 consecutive days. Mice were then maintained on a 12:12 LD cycle in wheel cages for 15 days prior to release into DD. *(E)* Representative diurnal activity profiles during LD are shown. *(F-G)* Quantitative RT-PCR analysis of *(F) IKKβ* and *Per2* mRNA in *CAGGCre-ER; IKKβ*$^{fx/fx}$ and control littermate (*IKKβ*$^{fx/fx}$, *CAGGCre-ER*, and WT) mice which received either oil or tamoxifen (n=3-5) and *(G) IKKβ* and panel of core clock genes in white adipose tissue (WAT) and hypothalamus of *CAGGCre-ER; IKKβ*$^{fx/fx}$ mice 5 weeks following either tamoxifen or oil injection (n=5-9). *(H-I)* Quantitative RT-PCR analyses of PAR bZIP genes in *(H)* liver or WAT of saline- or LPS-treated WT mice (n=5) or *(I)* liver of oil- or tamoxifen-treated *CAGGCre-ER; IKKβ*$^{fx/fx}$ mice (n=10-11). Data are represented as mean ± SEM. *$p \leq 0.05$, **$p \leq 0.01$, ***$p \leq 0.001$ unpaired t test.

Figure S4. High fat diet represses clock repressors. *(A)* Representative UCSC genome browser images of p65 ChIP-seq tracks at the core clock activator genes *Clock* and *Bmal1* in RC and HFD. Normalized tag counts indicated on y-axis. Orientation for each gene is indicated below each browser track. *(B)* Quantitative RT-PCR analysis of core clock genes in liver and white adipose tissue of mice at ZT8 following either RC (black) or HFD (orange) feeding (n=5-11). Data are represented as mean $\pm$ SEM. *$p \leq 0.01$, **$p \leq 0.01$, ***$p \leq 0.001$ *(C)* KEGG pathways analysis reveals CLOCK/BMAL1 enrichment at both lipid metabolism and immune pathways following high fat feeding.

Figure S5. p65 binds core clock genes across multiple cell types. Comparative analysis of GEO repository NF-κB p65 ChIP-seq data for TNFα-stimulated human lymphoblasts, human umbilical vein endothelial cells (HUVECs), HELA B2, and A549 lung carcinoma cells. Representative

ChIP-seq browser tracks depict NF-κB p65 binding in the promoter regions of the canonical circadian clock repressor genes (*Per1, Per2, Rev-erb*α) in each of these cell types. Vertical green tick marks indicate locations of predicted E-box motifs that aligned with the p65 peaks (Nakahata et al. 2008; Heinz et al. 2010).

Table S1. Macronutrient composition of high fat diet. Nutrient composition (% kcal) of the regular chow diet (RC, Harlan Teklad 7102) compared to the high fat diet (HFD, Research Diet, Inc, Custom Diet # D06022405) which was custom made (*) in this study.

Table S2. Primer names and sequences for quantitative real-time PCR used in this study.

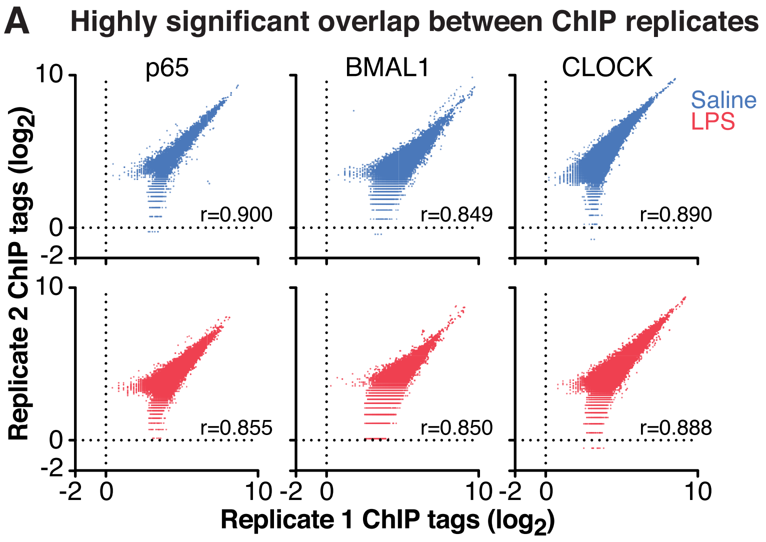

B Percentage of peaks containing consensus motif sequence

Motif name	Saline (% total sites)	LPS (% total sites)
NFκB-p65/NFκB-p50	7.9	44.7
Ronin-Thap11/GFY	31.3	9.3
ZNF143	35.9	13.7
CEBP	15.2	29.6
STAT	18.3	26.8
HNF6	8.1	12.3
FOXA	14.5	22.8
JUN-AP1	5.1	11.7
E-box	21.5	21.9
Total number of sites	3535	12052

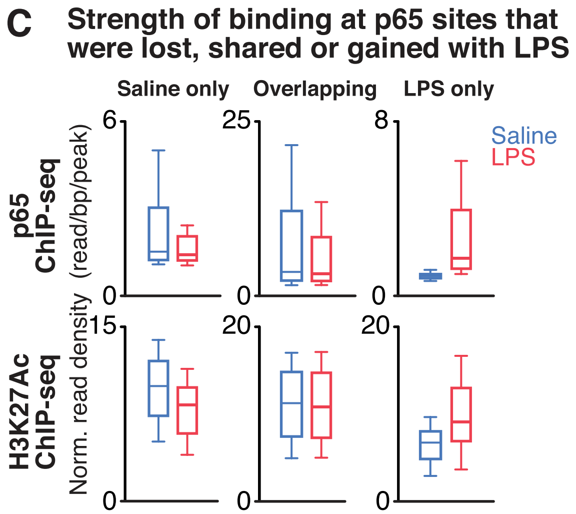

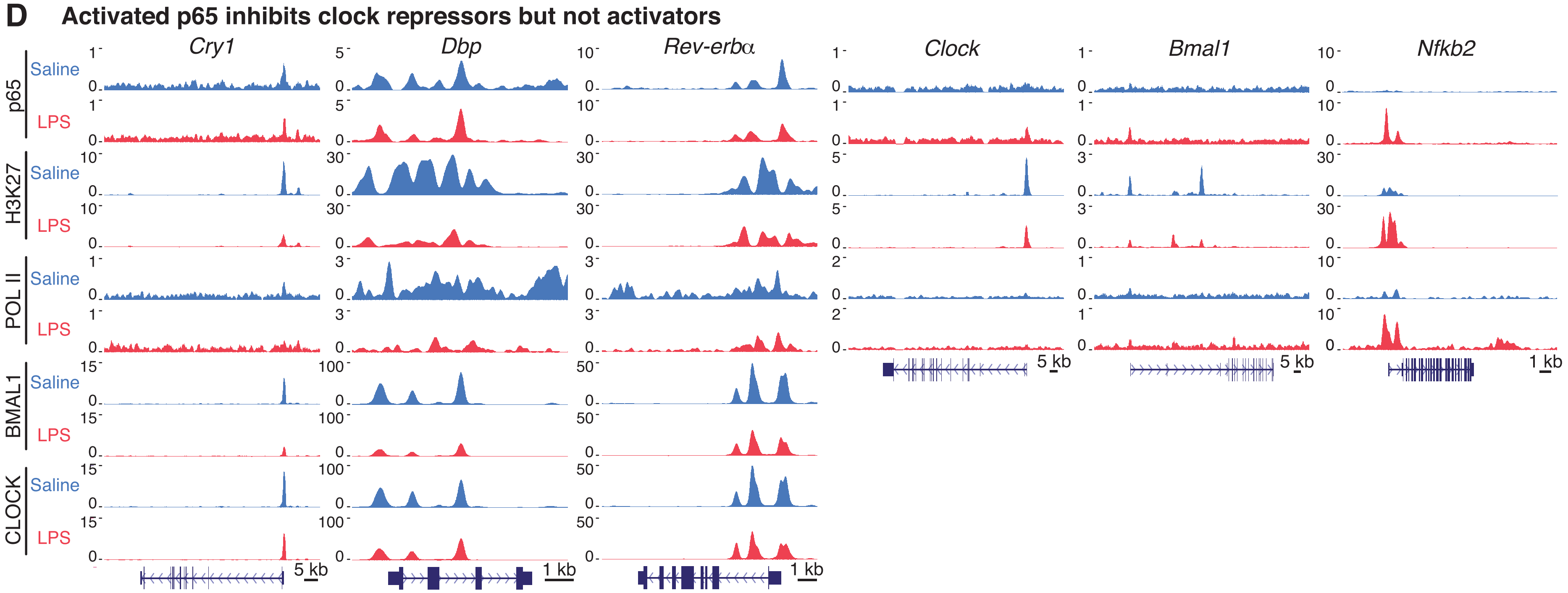

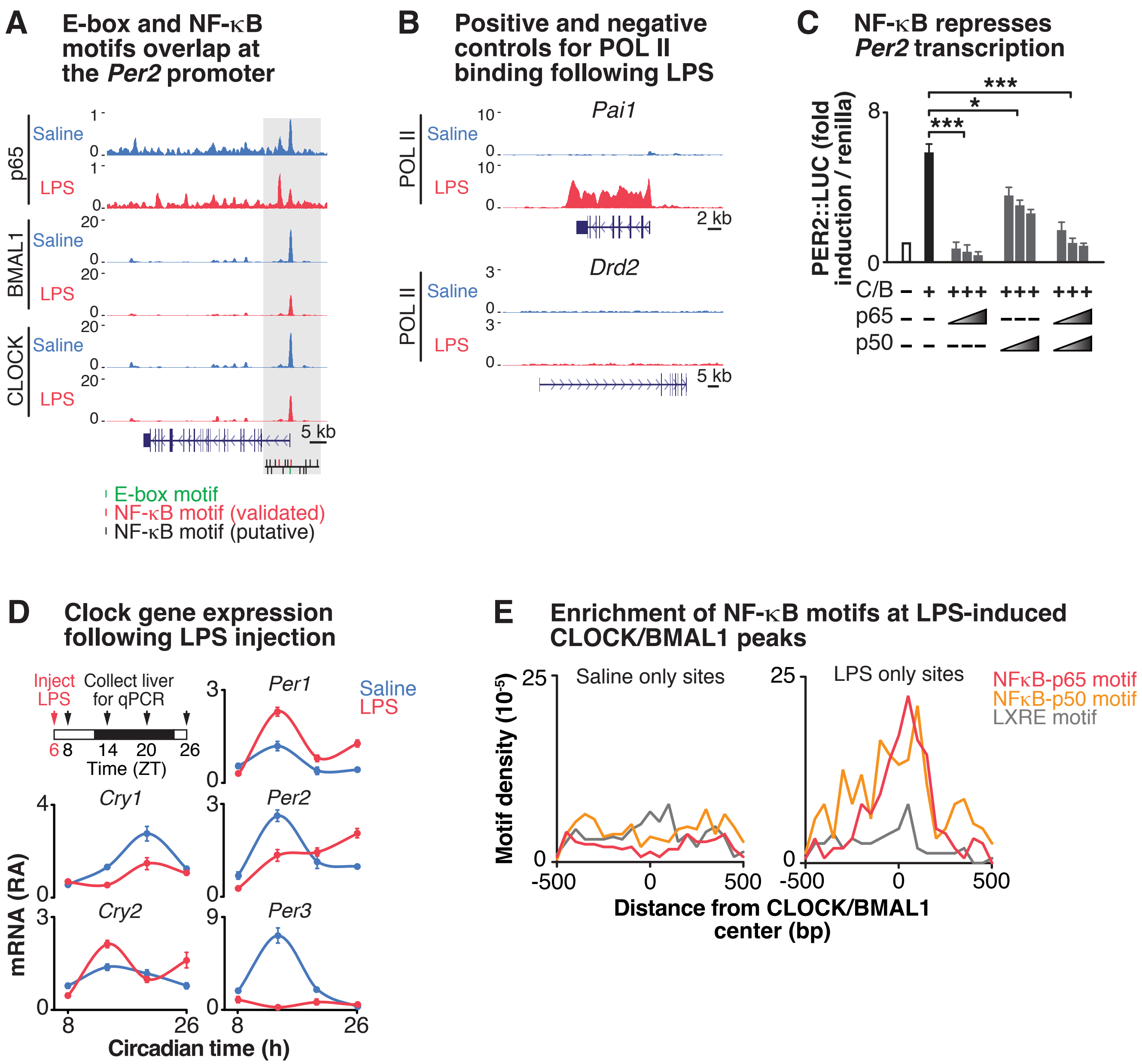
A
E-box and NF-κB motifs overlap at the Per2 promoter
p65
Saline
LPS
BMAL1
CLOCK
5 kb
E-box motif
NF-κB motif (validated)
NF-κB motif (putative)
B
Positive and negative controls for POL II binding following LPS
Pai1
Drd2
POL II
Saline
LPS
2 kb
5 kb
C
NF-κB represses Per2 transcription
PER2::LUC (fold induction / renilla)
***
*
***
C/B
p65
p50
D
Clock gene expression following LPS injection
Inject LPS
Collect liver for qPCR
Time (ZT)
Per1
Saline
LPS
Cry1
Per2
Cry2
Per3
mRNA (RA)
Circadian time (h)
E
Enrichment of NF-κB motifs at LPS-induced CLOCK/BMAL1 peaks
Saline only sites
LPS only sites
NFκB-p65 motif
NFκB-p50 motif
LXRE motif
Motif density (10^{-5})
Distance from CLOCK/BMAL1 center (bp)

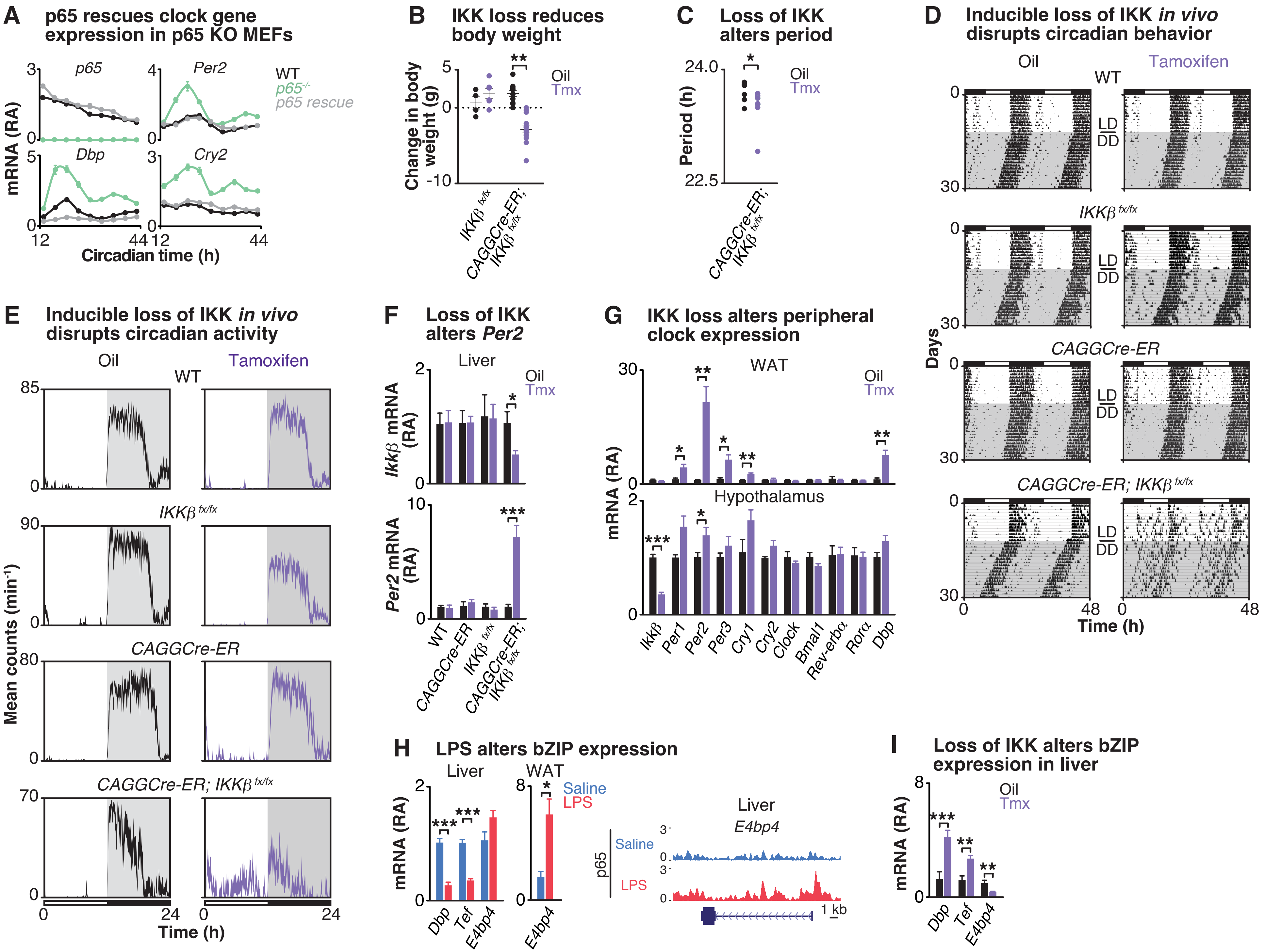
A p65 rescues clock gene expression in p65 KO MEFs
p65
Per2
Dbp
Cry2
WT
p65-/-
p65 rescue
mRNA (RA)
Circadian time (h)
B IKK loss reduces body weight
Change in body weight (g)
Oil
Tmx
IKKβ fx/fx
CAGGCre-ER; IKKβ fx/fx
C Loss of IKK alters period
Period (h)
Oil
Tmx
CAGGCre-ER; IKKβ fx/fx
D Inducible loss of IKK in vivo disrupts circadian behavior
Oil
Tamoxifen
WT
LD
DD
IKKβ fx/fx
CAGGCre-ER
CAGGCre-ER; IKKβ fx/fx
Days
Time (h)
E Inducible loss of IKK in vivo disrupts circadian activity
Oil
Tamoxifen
WT
IKKβ fx/fx
CAGGCre-ER
CAGGCre-ER; IKKβ fx/fx
Mean counts (min-1)
Time (h)
F Loss of IKK alters Per2
Liver
Oil
Tmx
Ikkβ mRNA (RA)
Per2 mRNA (RA)
WT
CAGGCre-ER
IKKβ fx/fx
CAGGCre-ER; IKKβ fx/fx
G IKK loss alters peripheral clock expression
WAT
Oil
Tmx
Hypothalamus
mRNA (RA)
Ikkβ
Per1
Per2
Per3
Cry1
Cry2
Clock
Bmal1
Rev-erbα
Rorα
Dbp
H LPS alters bZIP expression
Liver
WAT
Saline
LPS
mRNA (RA)
Dbp
Tef
E4bp4
Liver
E4bp4
p65
Saline
LPS
1 kb
I Loss of IKK alters bZIP expression in liver
Oil
Tmx
mRNA (RA)
Dbp
Tef
E4bp4

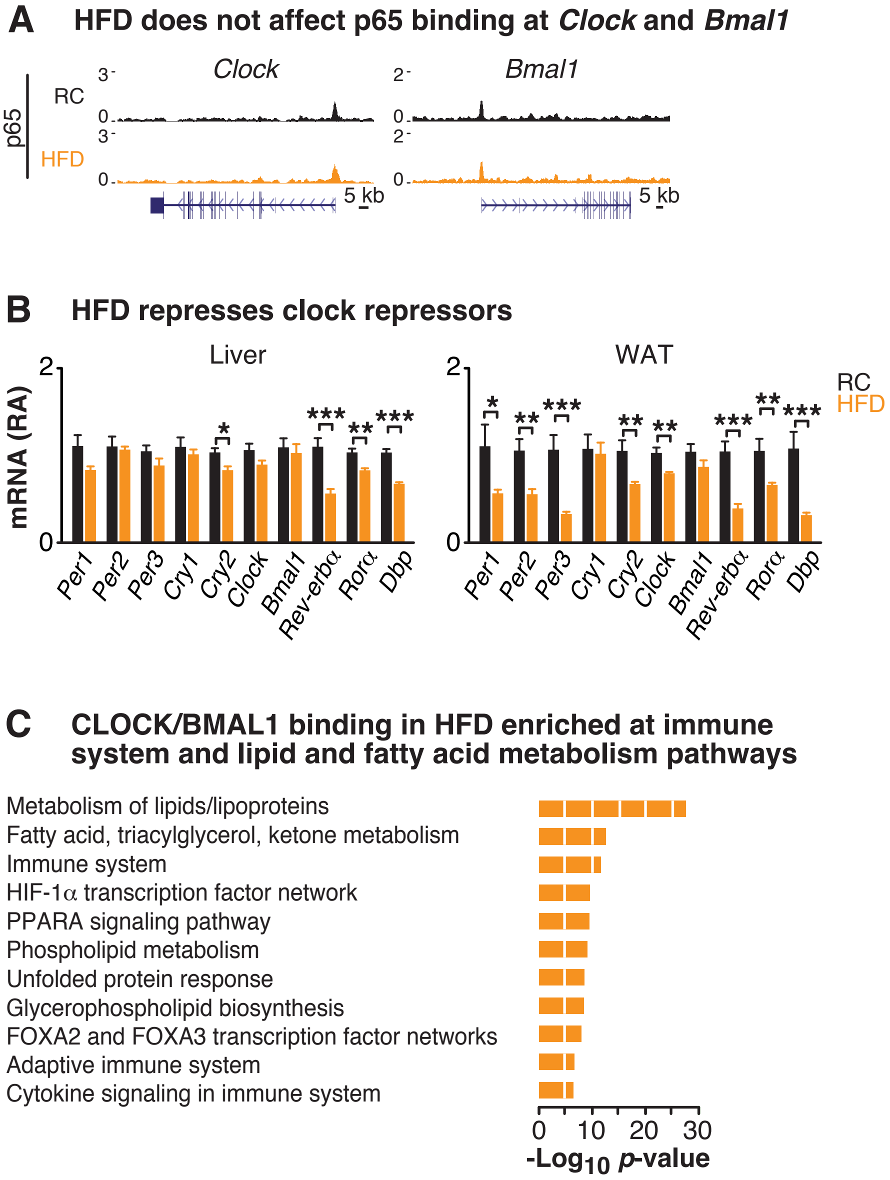
A
HFD does not affect p65 binding at *Clock* and *Bmal1*
Clock
Bmal1
p65
RC
HFD
3
0
2
5 kb
B
HFD represses clock repressors
Liver
WAT
mRNA (RA)
2
0
RC
HFD
Per1
Per2
Per3
Cry1
Cry2
Clock
Bmal1
Rev-erbα
Rorα
Dbp
C
CLOCK/BMAL1 binding in HFD enriched at immune system and lipid and fatty acid metabolism pathways
Metabolism of lipids/lipoproteins
Fatty acid, triacylglycerol, ketone metabolism
Immune system
HIF-1α transcription factor network
PPARA signaling pathway
Phospholipid metabolism
Unfolded protein response
Glycerophospholipid biosynthesis
FOXA2 and FOXA3 transcription factor networks
Adaptive immune system
Cytokine signaling in immune system
0
10
20
30
-Log10 *p*-value

p65 binds core clock repressors across multiple cell types

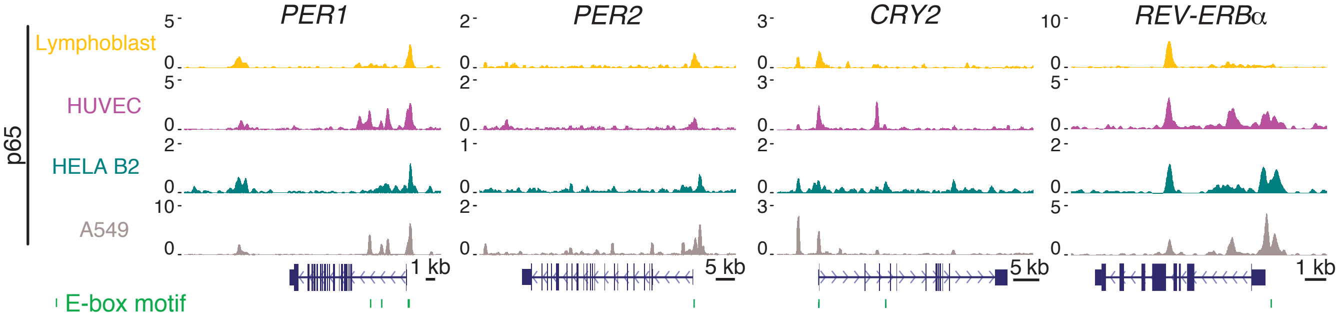

Kcal (%)	RC	HFD*
Protein	25	20
Carbohydrate	58	35
Fat	17	45
Saturated fatty acids (C4:0, C6:0, C8:0, C10:0, C12:0 C14:0, C16:0. C18:0, C20:0)	4.4	26.3
Monounsaturated fatty acids (C14:1, C16:1, C18:1, C20:1)	2.7	13.5
Polyunsaturated fatty acids (C18:2, C18:3)	9.9	5.2
Total kcal/g	3.1	4.73

Primer name	Primer sequence
Per1 F	CCCAGCTTTACCTGCAGAAG
Per1 R	ATGGTCGAAAGGAAGCCTCT
Per2 F	TGTGCGATGATGATTCGTGA
Per2 R	GGTGAAGGTACGTTTGGTTTGC
Per3 F	GTGATTGTTCACGCGTCTGT
Per3 R	CACTGCCATCTCGAGTTCAA
Cry1 F	TGAGGCAAGCAGACTGAATATTG
Cry1 R	CCTCTGTACCGGGAAAGCTG
Cry2 F	CTGGCGAGAAGGTAGAGTGG
Cry2 R	GACGCAGAATTAGCCTTTGC
Clock F	ACCACAGCAACAGCAACAAC
Clock R	GGCTGCTGAACTGAAGGAAG
Bmal1 F	AGGCCCACAGTCAGATTGAA
Bmal1 R	TGGTACCAAAGAAGCCAATTCAT
Rev-erbα F	CAGTGCTCCTGGCATGACTA
Rev-erbα R	CCACATCCCCACAGACTTTA
Rorα F	ACGCCCACCTACAACATCTC
Rorα R	TGCCCATCCATATAGGTGCT
Dbp F	GCAGAGTCCTGTTCCTTGCT
Dbp R	CTGCATCATGACGTTCTTCG
Tef F	ATCTTTCAGCCCTCGGAAAC
Tef R	GGTCTCCCTCTCCTTTTCCA
E4BP4 F	CGGAGCTTGAATCGCGCCCC
E4BP4 R	GGGTTATCGTGGTTCTGCTCCCTG
Ikkβ F	CAGCCCAAAGAACAGAGACC
Ikkβ R	ACCACATTGGGATGGTTCAG
p65 F	ATCTTCTTGCTGTGCGACAA
p65 R	TGGTCCCGTGAAATACACCT
Gapdh F	CAAGGAGTAAGAAACCCTGGACC
Gapdh R	CGAGTTGGGATAGGGCCTCT
